# Dietary Practice among Type 2 Diabetic Ppatients in Southern Ethiopia

**DOI:** 10.1155/2021/1359792

**Published:** 2021-12-27

**Authors:** Kidus Temesgen Worsa, Beakal Zinab, Melese Sinaga Teshome

**Affiliations:** ^1^School of Public Health, College of Health Science, Arba Minch University, Arba Minch, Ethiopia; ^2^Department of Nutrition and Dietetics, Faculty of Public Health, Health Institute, Jimma University, Jimma, Ethiopia

## Abstract

**Background:**

Diabetic patients' dietary practice is critical to improve glycemic, lipid, and blood pressure control. However, a significant number of patients had poor dietary practice. In Ethiopia, more than half of diabetic patients were not practicing a healthy dietary approach. Therefore, this study assessed variables that were hardly addressed in previous studies. The aim of this study was to assess dietary practice and associated factors among patients with type 2 diabetes.

**Methods:**

A facility-based cross-sectional study was performed among patients with type 2 diabetes in Arba Minch General Hospital from April 21 to May 20, 2020. A systematic sampling technique was used to select 352 patients. The data were entered into EpiData version 3.1 and exported to SPSS version 21 for cleaning and analysis. Descriptive statistics were performed. All variables in bivariate analysis with *p*-value <0.25 were entered into a multivariable logistic regression model, and statistical significance was declared at a *p*-value of less than 0.05.

**Results:**

The prevalence of poor dietary practice was found to be 40.6% (95%CI (35.7–46.0)). After adjusting for other variables in multivariable analysis, not attending formal education (AOR = 3.0; 95%CI (1.6–5.5)), being at primary education level (AOR = 2.2; 95%CI (1.1–4.4)), being moderately food insecure (AOR = 5.3; 95%CI (2.8–9.9)), having depression (AOR = 5.9; 95%CI (3.0–11.4)), and not having nutrition education (AOR = 2.2; 95% (1.1–4.6)) were factors associated with poor dietary practice.

**Conclusions:**

A significant proportion of patients had poor dietary practice. The poor dietary practice was significantly higher among those with no formal education, at the primary education level, from the moderately food-insecure household, having depression, and not having nutrition education. The results imply the need for strengthening health information dissemination concerning healthy dietary practice in the form of a package.

## 1. Introduction

Type 2 diabetes accounts for 90–95% of those with diabetes, previously referred to as noninsulin-dependent diabetes or adult-onset diabetes and encompasses individuals who have insulin resistance and usually have relative (rather than absolute) insulin deficiency. It is a metabolic disorder manifested by increased blood glucose level and caused by a complex interaction of genetics and environmental factors. Diabetic patients usually experience acute, chronic, or both complications. Acute complications of poorly managed diabetes are hyperglycemia and hypoglycemia. The chronic complications are associated with long-standing impairment, malfunction, and failure of different organs, especially the eyes, kidneys, nerves, heart, and blood vessels [1–3].

Dietary shifts almost concurrently occur with demographic and epidemiologic shifts. Motorized ways of life and mechanization contribute to an increased prevalence of nutrition-related noncommunicable diseases. There are limited pieces of evidence on effective strategies to prevent the onset of poor nutritional status in low- and middle-income countries (LMICs) [4–6].

Globally, in 2019, half a billion people are estimated to be living with diabetes, representing 9.3% of the global adult population. This figure will increase to 578 million (10.2%) in 2030 and 700 million (10.9%) in 2045 [7]. The percentage of premature deaths attributable to diabetes is higher in low- and middle-income countries than in high-income countries [8].

Nutrition is crucial in the prevention or delay and management of type 2 diabetes including those already on medications. The primary goals of nutrition therapy for diabetes are to improve glycemic, lipid, and blood pressure control, thus contributing to reduced risk for potential long-term complications of diabetes and heart disease, and to improve the quality of life of individuals with diabetes. A diet that is diverse in food groups is independently associated with a lower risk of type 2 diabetes. People who reported consuming a diversified diet had a 30% reduced incidence of type 2 diabetes. Low-calorie eating patterns, specifically higher intakes of nuts, berries, yogurt, coffee, and tea, are associated with reduced diabetes risk. On the other hand, red meats and sugar-sweetened beverages are associated with a high risk of type 2 diabetes [8–12].

Mortality and morbidity associated with noncommunicable disease (NCD) surprisingly increased in low- and middle-income countries. In 2016, 78% of all NCD deaths and 85% of premature adult NCD deaths happened in low- and middle-income countries (LMICs) [13–15]. This burden is more observed in the working age (15–64 years of age) of the regions [16]. In Africa, the prevalence of diabetes increased from 3.1% (4 million) in 1980 to 7.1% (25 million) in 2014 [17]. With this trend, Africa is out of track in realizing the NCD pointers from the set global and continental target [18]. In Ethiopia, NCD alone was responsible for 39% of all deaths, and out of which, diabetes attributed to 2% of all deaths. Tobacco use, poor diet, harmful use of alcohol, and obesity were dominant risk factors for the causation of NCD [15].

Diabetic patients' dietary practice is the key to the management of type 2 diabetes and the prevention of complications related to hyperglycemia [12, 19]. However, the patients' dietary practice was poor and underemphasized in the diabetes management component [20].

There were extensive works that had been performed by governments of low-income countries addressing the increasing prevalence of type 2 diabetes via various health strategies and policies. The second Ethiopian National Nutrition Program (NNP-II) has given a place for NCDs through numerous initiatives. However, the burden is still high [21, 22]. In sub-Saharan Africa (SSA), diabetic patients hardly practice the advised diet and also 33% to 87% of patients had a poor level of knowledge about diabetes-related complications [23]. As a result, sugar-sweetened beverages (SSB) were highly consumed and pointedly contributed to total sugar and energy consumption in SSA [21].

In Ethiopia, diabetes is a major public health problem in the contemporary era. Concomitantly, more than half of diabetic patients were not practicing the perceived healthy dietary approach. Some of the identified factors were eating out of home, attending diabetic nutrition education, and having the disease for more than 10 years [24]. Additionally, facing difficulty to choose foods, nonavailability of fruits and vegetables, and worrying about the high cost of foods were the factors significantly associated with poor dietary practice [25].

It was agreed that dietary management had a central role in diabetic management, but little is known about the dietary practice and associated factors in Ethiopia, in general, and in the study area, in particular, and also very crucial variables were not addressed in the past studies regarding their dietary practice. Therefore, this study assessed variables that were hardly addressed in previous studies performed both in Ethiopia and particularly the study area, concerning dietary practice among patients with type 2 diabetes.

## 2. Methods and Materials

### 2.1. Study Area and Period

The study was conducted from April 21 to May 20, 2020, in Arba Minch General Hospital. Arba Minch is the capital town of Gamo Zone located 505 km far to the south of Addis Ababa, the capital city of Ethiopia and 272 km from Hawassa, center of Southern Nations, Nationalities, and People's Regional State (SNNPR). The town is one of the low-land areas in the SNNPR having a hot climate with an average temperature of 29°c and annual mean rainfall of 900 mm. An estimated population of 128,721 was living in Arba Minch town. There was one general hospital, one primary hospital, and two health centers currently proving health service [26]. The area is well known for its fruit production such as banana, mango, apple, avocado, orange, papaya, and vegetables like moringa, cabbage, and carrot. Additionally, the area is also familiar with the production and consumption of fish [27–29].

### 2.2. Study Design

A facility-based cross-sectional study was employed.

### 2.3. Source Population

All adults diagnosed with type 2 diabetes mellitus were on regular follow-up at Arba Minch General Hospital, diabetes follow-up clinic.

### 2.4. Study Population

Those selected adult patients with type 2 diabetes who had regular follow-up and fulfilled the inclusion criteria were considered as our study population. Adult patients with type 2 diabetes who had follow-up for at least six months at Arba Minch General Hospital were included in the study, whereas pregnant mothers and mentally ill patients who were unable to respond were excluded from the study.

### 2.5. Sample Size Determination and Sampling Procedure

The sample size was determined using a single population proportion formula. The assumptions considered were *p*=51.4%, the proportion of poor dietary practice taken from a study performed in Addis Ababa among patients with type 2 diabetes [25], 95% confidence interval, and 5% margin of error. Based on this assumption, the calculated sample size was 384. Because the source population was 2,000, which is less than 10,000, we used the population correction formula and the sample become 320. After considering the 10% nonresponse rate, the sample size required for the research was 352. A systematic random sampling technique was used.

### 2.6. Data Collection Tools

The dietary practice was assessed by using the modified form of the nineteen-item Morisky medication adherence scale adapted from different literatures [25, 30, 31]. Questions about knowledge on diabetes were adapted from literature [32]. Questions about household food insecurity were measured using the Food and Nutrition Technical Assistance (FANTA) tool called Household Food Insecurity Access Scale (HFIAS) [33]. The respondents were asked about the occurrence of the condition that was whether the condition in the question happened at all in the past four weeks (yes or no). For respondents who answered “yes” to the occurrence question, the frequency of occurrence of the condition was asked to determine whether the condition rarely happened (once or twice), sometimes (three to ten times), or often (more than ten times) in the past four weeks. Mental- and behavior-related questions were developed by adapting and modifying from different literatures [34, 35]. Depression and anxiety status were assessed by using the Hospital Anxiety and Depression Scale (HADS), and the items rated on a four-point scale range from 0 to 3 [36–38].

### 2.7. Measurement

Anthropometric measurements were taken using standardized techniques and calibrated equipment. Height was measured using a stadiometer at the Frankfurt plane; participants stood in an erect posture with their shoulder level and hand at their side, thighs and heels together at a comfortable position, buttocks, scapula, and head positioned in contact with a vertical stand of stadiometer. During the measurement, respondents took off their shoes and the result was recorded to the nearest 0.1 cm [39]. Weight was measured three times by digital scale (Seca Germany), to the nearest 0.1 kg in light indoor clothing with bare feet. The scale was calibrated to zero before and after each measurement. Body mass index (BMI) was calculated as BMI: body weight (kg)/body height (m^2^). Waist circumference (WC) was measured midway between the inferior angle of the 10th ribs and the iliac crest at the end of normal expiration to the nearest 1 cm using a nonstretchable rubber measuring tape, participants in an upright position, with arms relaxed at the side, and feet evenly spread apart [39, 40]. Nutritional status was categorized into 4 groups; underweight with BMI < 18.50 kg/m^2^, normal if BMI 18.50–24.99 kg/m^2^, overweight if BMI 25–29.99 kg/m^2^, and obese if BMI ≥ 30 kg/m^2^. Central obesity was defined by waist circumference of >102 cm for men and >88 cm for women [39, 41].

### 2.8. Data Processing and Analytical Procedures

The data were entered into EpiData software version 3.1 and then exported to SPSS window version 21 for data cleaning and analysis. Descriptive statistics including frequency, percentages, and summary measures (mean and standard deviation) were performed. Fulfillment of chi‐square assumption was checked to select variables for logistic regression. Bivariate logistic regression was performed to select candidate variables for multivariable binary logistic regression. All variables that had *p*-value <0.25 in bivariate analysis were entered into the multivariable logistic regression model to assess the association between dependent and independent variables. The odds ratio was used to measure the degree of association.

Principal component analysis (PCA) was performed for the household wealth index after checking the assumptions for PCA. In the final iteration step, the factor score was taken and ranked into tertials. Dietary practice status score was computed by taking the mean score of 19 questions and labeled them as good and poor dietary practice for above and below the mean value, respectively. Family support was calculated by summing the scores of 12 items and obtaining the mean of the summation, and finally the mean score was dichotomized to yes or no alternatives for the presence and absence of family support, respectively. Patients' knowledge about diabetes was similarly calculated by taking the mean values of 24 questions and labeling as good and poor knowledge for above and below the mean value, respectively.

Food security status was computed by using the HFIAS occurrence, and frequency questions and Insecurity Access Scale score were analyzed based on the HFIAS criteria and categorized into food secured, mildly food insecure, moderate food insecure, and sever food insecure [33].

Anxiety was computed by taking the sum of seven questions and labeled as yes and no for the presence and absence of anxiety with greater than or equal to 8 and less than 8, respectively. Depression was computed by taking the sum of seven questions and labeled as yes and no for the presence and absence of depression with greater than or equal to 8 and less than 8, respectively. Multicollinearity was checked by variance inflation factors, and the goodness-of-fit model by Hosmer–Lemeshow for *p*-value > 0.05 was checked. A multivariable logistic regression model was used for factors associated with the dietary practice, and statistical significance was defined at a *p*-value of less than 0.05. Data quality was assured by the proper designing of the questionnaire. The questionnaire was pretested on 5% of the sample size in the nearby hospital, Chencha Hospital.

Cronbach's alpha was checked for wealth index and dietary practice with the value of 0.85 and 0.74, respectively. Data collectors were trained for one day on how to conduct the data collection and supervision, respectively, and for experienced data collector's priority was given. After completion of the data collection, each questionnaire was checked for completeness and consistency on a daily basis. Anthropometric measurements were standardized by giving training for data collectors, and instruments were calibrated each day. Before the study began, ethical clearance was obtained from the ethical review committee of the Jimma University with a reference number of IRB 00060/2020. Official permission was secured from Arba Minch General Hospital. Participants provided written consent before the interviews. Confidentiality was ensured throughout the process of the study. The consent of each participant was taken by the data collectors and kept in their respective questionnaire and confidentially kept.

## 3. Result

### 3.1. Sociodemographic and Economic Characteristics

More than half of the respondents, 197 (56.0%), were men. More than two-thirds of the respondents (72.4%) were, married and nearly half (49.1%) of participants were secondary and above educational status. About one-third (33.2%) of participants were government employees. Similarly, about one-third (33.24%) of the participants were found in the lowest wealth category. Regarding family support, two hundred forty-one (68.5%) respondents had no had family support (Table 1). Among respondents that reside in households with moderate food insecurity, more than half (57.7%) were practicing a poor diet.

### 3.2. Health- and Treatment-Related Characteristics of Patients with Type 2 Diabetes

More than half of respondents (54.8%) were diagnosed with the disease less or equal to 5 years back. About 39.2% of the respondents had comorbidity. Regarding the medication regimen, more than two-thirds (69.3%) of the respondents were on oral medication and 27 (7.7%) were on both oral and insulin injection. Nearly two-thirds (64.8%) of the respondents regularly attended diabetic education. Most of the respondents (88.6%) received nutrition education related to diabetes. Regarding body mass index, 34.9%, 38.1%, and 23.9% of the respondents were normal, overweight, and obese, respectively. Likewise, 71.3% had central obesity. More than half (51.7%) of the respondents were members of the Ethiopian Diabetes Association. Most (82.1%) of the respondents had inadequate glycemic control (Table 2).

### 3.3. Mental- and Behavior-Related Characteristics of Patients with Type 2 Diabetes

Regarding alcohol consumption, forty-four (12.5%) respondents used to drink alcohol, and eight (2.3%) of the respondents were smokers. Similarly, 2.3% of the respondents reported that they chewed khat. According to this study, there was no diabetes-related food taboo among the respondents. Seventy-eight (22.2%) respondents had experienced anxiety. Among the respondents, ninety-five (26.9%) had experienced depression (Table 3).

### 3.4. Dietary Practice and Other Related Characteristics

Among the respondents, the majority (82.4%) did not consume food outside their home. Two hundred ninety-five (83.8%) respondents answered that vegetables were available in their area. From the respondents, fifty (14.2%) respondents had faced difficulty to choose foods. More than one-third (34.1%) of the respondents had a fasting history before the diagnosis of diabetes, and seventy-two (20.5%) respondents had fasted after the diagnosis of diabetes. More than half (57.7%) of the respondents had poor knowledge about diabetes. This study indicated that two hundred nine (40.6%, 95%CI (35.76, 46.0)) of respondents had poor dietary practice (Table 4).

### 3.5. Factors Associated with Dietary Practice of Patients with Type 2 Diabetes

Bivariate and multivariable logistic regression analysis was performed to identify factors associated with the dietary practice of patients with type 2 diabetes. On the bivariate analysis, educational status, residence, household food security status, duration of diabetes, diabetic knowledge, despondency, depression, and nutrition education showed a *p*-value of <0.25 and became a candidate for multivariable analysis. On multivariable analysis, by taking other variables as constant, patients who had not attended formal education and those at primary education level were 3.0 and 2.2 times more likely to have poor dietary practice as compared to secondary education and above (AOR = 3.0; 95%CI (1.6–5.5)) and (AOR = 2.2; 95%CI (1.1–4.4)), respectively.

Participants from the moderately food-insecure households were 5.3 times more likely to have poor dietary practice as compared to food-secure households (AOR = 5.3; 95%CI (2.8–9.9)). Those patients having depression were 5.9 times more likely to have poor dietary practice than the counter ones (AOR = 5.9; 95%CI (3.0–11.4)). Patients who did not get nutrition education were 2.2 times more likely to have poor dietary practice compared to those who get nutrition education (AOR = 2.2; 95%CI (1.1–4.6)) (Table 5).

But residence, duration of knowledge about diabetes, and patient's despondency were not statistically associated with poor dietary practice (Table 5).

Hosmer–Lemeshow's goodness-of-fit test produces the chi-square of 2.962 with *p* value of 0.94 and 8 degrees of freedom; hence, the model was good for the data.

## 4. Discussion

The findings of the present study revealed that a significant proportion (40.6%) of diabetic patients had poor dietary practice. The finding is higher than previous reports from Kenya, Nigeria, and Yemen, which were 26.1%, 24.0%, and 32.4%, respectively [42–44]. The disparity might be attributed to the sociodemographic and economic domains of the study participants. This might be because of the different assessment methods used to assess the level of dietary practice in those studies. Additionally, in the current study substantial proportion of patients (42.3%) had poor knowledge regarding diabetes which might have made them to consume monotonous and undiversified diets. In the present study, almost one-fifth of patients had eaten out of home, which might hinder the patients' dietary practice to step back. Concurrently, in this study huge proportion of individuals had no family support which may play a great role in poor dietary habits.

The finding of this study was in line with studies performed in Botswana, Pakistan, and Jimma, which showed that the prevalence of poor dietary practice was 37.0%, 36.1%, and 36%, respectively [42, 45, 46]. But in some other studies from Ethiopia and elsewhere, the proportion of poor dietary practice was much higher than this study [25, 30, 47–49]. The difference might be accounted for by differences in study period and measurement. Furthermore, the sedentary and motorized way of life in those areas might increase exposure to the consumption of fast and energy-dense foods, which might subsequently make the patients' dietary practice to be more deprived [50].

This study also showed that patients' educational status had a significant association with dietary practice. Patients who had not attended formal education and being at primary education were more likely to have poor dietary practice as compared with college graduates and above, respectively. The finding was consistent with a study conducted in Kenya and Bahir Dar [30, 51]. This might be because the patients' educational status had a direct relation with their dietary practice. Patients with higher educational status would likely give due attention to a healthy and quality diet because education gives an opportunity for patients to have better health literacy, which may in turn improve dietary practice. Moreover, those with lower education might have a limited chance of getting dietary information [52].

According to this study, the poor dietary practice was higher among patients residing in food-insecure households. Similarly, a study performed in the United States of America (USA) showed that food insecurity had a negative effect on patients' dietary habits [53]. It is apparent that patients from food-insecure households would likely have economic constraints that might adversely affect the household's access to diversified food. Likewise, evidence indicated that less expensive and calorie-dense food consumption among food-insecure households might also play a crucial role [54, 55]. Additionally, patients might be engaged in negative coping strategies to tackle the food insecurity situation, which in turn might play a key role in their poor dietary practice [56, 57]. Furthermore, 14.2% of the patients in this study faced difficulty to choose foods. This might be because of a limited resource since one-third of the patients in this study were in a wealth index of the lower category.

The findings of this study indicated that patients who had depression were more likely to have poor dietary practices than those having no depression. Similarly, a finding from Ghana, Saudi Arabia, and the Republic of Korea showed that depression status was higher among patients with poor dietary habits [58–60]. Depression might pose an adverse effect on a patient's decision-making ability to their general health and toward wise food choices and self-care [61, 62]. Additionally, the association might be because of less preference for social interaction of depressed patients, and they might not disclose their medical history and seek social support from their family. In this study, more than two-thirds (68.5%) of patients had no family support. Also, more than one-third of patients had comorbidities other than diabetes that could create another burden for the patients and might have played a role in affecting healthy dietary habit in the study area.

In this study, the poor dietary practice was higher among diabetic patients who had not received nutrition education. The finding is consistent with a study conducted in Bahir Dar, Northwest Ethiopia [25], but was not in agreement with a study performed in the capital, Addis Ababa. It is evident that the dissemination of appropriate information is vital and the first step to make healthy choices. A study performed in Kenya reported that good dietary practice and higher dietary diversity among patients were associated with better nutrition knowledge [51]. The result of this study implied that diabetic patients' dietary practice was undesirably colossal. As a result, there is a need to strengthening of healthy diet-centered diabetic care and focusing on the identified factors for the poor dietary practice in the study area.

This study considered new variables like depression and food insecurity in relation to dietary practice that were, to the best of our knowledge, not assessed in other previous studies performed so far. Using a small sample size for the study and employing a cross-sectional study design were limitations of this study. Additionally, recall bias might have been there in the tool used to obtain data about household food insecurity and social desirability bias might be introduced for questions assessing diabetic knowledge. As a result, interpretation of the findings of this study needs caution.

## 5. Conclusions

The proportion of poor dietary practice among patients with type 2 diabetes on follow-up attending at Arba Minch General Hospital was high. Patients' educational status, household food security status, depression status, and receipt of nutrition education were statistically significantly associated with their poor dietary practice. Health information dissemination concerning healthy dietary practices should be strengthened and delivered in the form of a package. Health professionals should have a common understanding and follow patients' adherence to the dietary recommendation and should also deliver patient-oriented nutritional education in every follow-up visit.

## Figures and Tables

**Table 1:** Sociodemographic and economic characteristics of diabetic patients attending follow-up at Arba Minch General Hospital, South Ethiopia, 2020 (*n* = 352).

Variables	Categories	Frequency (*n* = 352)	Percent

Age	<40	75	21.3
	40–60	211	59.9
	>60	66	18.8

Sex	Male	197	56.0
	Female	155	44.0

Residence	Urban	287	81.5
	Rural	65	18.5

Marital status	Single	26	7.4
	Married	255	72.4
	Widowed	33	9.4
	Divorced	38	10.8

Educational status	No formal education	106	30.1
	Primary education	73	20.7
	Secondary and above	173	49.1

Occupation	Unemployed	133	37.8
	Government employed	117	33.2
	Private employed	102	29.0

Household wealth	Low	117	33.24
	Middle	118	33.52
	High	117	33.24

Family support	Yes	111	31.5
	No	241	68.5

Food security	Secure	128	36.4
	Mild	120	34.1
	Moderate	104	29.5

**Table 2:** Health- and treatment-related characteristics of diabetic patients attending follow-up at Arba Minch General Hospital, South Ethiopia, 2020 (*n* = 352).

Variables	Categories	Frequency (*n* = 352)	Percent

Duration of diabetes	≤5 year	193	54.8
	>5 year	159	45.2

Comorbidity	Yes	138	39.2
	No	214	60.8

Current medication	Insulin injection	74	21.0
	Oral medication	244	69.3
	Both	27	7.7
	Diet alone	7	2.0

Regularly attended a diabetic education	Yes	228	64.8
	No	124	35.2

Got nutrition education	Yes	312	88.6
	No	40	11.4

Despondency	Yes	184	52.3
	No	168	47.7

Member of Ethiopian diabetic association	Yes	182	51.7
	No	170	48.3

Body mass index	Underweight	11	3.1
	Normal	123	34.9
	Overweight	134	38.1
	Obese	84	23.9

Central obesity	Normal	101	28.7
	Obese	251	71.3

Members of Ethiopian diabetic association	Yes	182	51.7
	No	170	48.3

Glycemic control	Adequate glycemic control	63	17.9
	Inadequate glycemic control	289	82.1

**Table 3:** Substance using status of diabetic patients attending follow-up at Arba Minch General Hospital, South Ethiopia, 2020 (*n* = 352).

Variables	Categories	Frequency (*n* = 352)	Percent

Ever drunk alcohol	Yes	44	12.5
	No	308	87.5

Currently alcohol drinking (*n* = 44)	Yes	32	72.7
	No	12	27.3

Ever smoked cigarette	Yes	8	2.3
	No	344	97.7

Currently smoking cigarette (*n* = 8)	Yes	3	37.5
	No	5	62.5

Ever chewed khat	Yes	8	2.3
	No	344	97.7

Currently chewing khat (*n* = 8)	Yes	4	50.0
	No	4	50.0

Depression	No depression	257	73.1
	Have depression	95	26.9

Anxiety	No anxiety	274	77.8
	Anxiety	78	22.2

**Table 4:** Dietary-related characteristics of diabetic patients attending follow-up at Arba Minch General Hospital, South Ethiopia, 2020 (*n* = 352).

Variables	Categories	Frequency (*n* = 352)	Percent

Eating out of home	Yes	62	17.6
	No	290	82.4

Availability of vegetables	Yes	295	83.8
	No	57	16.2

Availability of fruits	Yes	291	82.7
	No	61	17.3

Faced difficulty in choosing food?	Yes	50	14.2
	No	302	85.8

Worry about high cost of foods	Yes	188	53.4
	No	164	46.6

History of fasting before diabetes	Yes	120	34.1
	No	232	65.9

History of fasting after diabetes	Yes	72	20.5
	No	280	79.5

Diabetic knowledge	Good	203	57.7
	Poor	149	42.3

Dietary practice	Poor	143	40.6
	Good	209	59.4

**Table 5:** Multivariable logistic regression model showing factors associated with the dietary practice of patients with type 2 diabetes in Arba Minch General Hospital, Southern Ethiopia, 2020 (*n* = 352).

Variables	Category	Dietary practice		COR (95%C.I)	AOR (95%C.I)	*p* value
		Poor (%)	Good (%)			

Educational status	No formal education	51 (48.1)	55 (51.9)	1.7 (1.1–2.7)	**3.0 (1.6–5.5)**	**0.001**
	Primary education	30 (41.1)	43 (58.9)	1.2 (0.7–2.2)	**2.2 (1.1–4.4)**	**0.030**
	Secondary and above	62 (35.8)	111 (64.2)	1	**1**	

Residence	Urban	112 (39.0)	175 (61.0)	1	1	
	Rural	31 (47.7)	34 (52.3)	1.4 (0.8–2.4)	1.6 (0.8–2.9)	0.171

Food security status	Secure	37 (28.9)	91 (71.1)	1	1	
	Mildly insecure	46 (38.3)	74 (61.7)	1.5 (0.9–2.6)	1.4 (0.8–2.6)	0.246
	Moderately insecure	60 (57.7)	44 (42.3)	3.4 (1.9–5.8)	**5.3 (2.8–9.9)**	**<0.001**

Duration of diabetes in years	≤5 year	87 (45.1)	106 (54.9)	1	1	
	>5 year	56 (35.2)	103 (64.8)	0.7 (0.4–0.9)	0.7 (0.4–1.1)	0.110

Diabetes knowledge	Good knowledge	76 (37.4)	127 (62.6)	1	1	
	Poor knowledge	67 (45.0)	82 (55.0)	1.4 (0.9–2.1)	1.7 (0.9–2.9)	0.061

Despondency	Yes	66 (35.9)	118 (64.1)	0.6 (0.4–1.1)	0.6 (0.4–1.1)	0.056
	No	77 (45.8)	91 (54.2)	1	1	

Depression	No depression	91 (35.4)	166 (64.6)	1	1	
	Have depression	52 (54.4)	43 (45.3)	2.2 (1.4–3.6)	**5.9 (3.0–11.4)**	**<0.001**

Nutrition education	Yes	121 (38.8)	191 (61.2)	1	**1**	
	No	22 (55.0)	18 (45.0)	2 (1.1–3.9)	**2.2 (1.1–4.6)**	**0.038**

## Data Availability

The datasets used and analyzed during the current study are included in the article.
